# Transcriptome Analysis Reveals Differential Gene Expression and a Possible Role of Gibberellins in a Shade-Tolerant Mutant of Perennial Ryegrass

**DOI:** 10.3389/fpls.2017.00868

**Published:** 2017-05-26

**Authors:** Wei Li, Lorenzo Katin-Grazzini, Xianbin Gu, Xiaojing Wang, Rania El-Tanbouly, Huseyin Yer, Chandra Thammina, John Inguagiato, Karl Guillard, Richard J. McAvoy, Jill Wegrzyn, Tingting Gu, Yi Li

**Affiliations:** ^1^Department of Plant Science and Landscape Architecture, University of Connecticut, StorrsCT, United States; ^2^College of Horticulture and State Key Laboratory of Crop Genetics and Germplasm Enhancement, Nanjing Agricultural UniversityNanjing, China; ^3^Department of Floriculture, Ornamental, Horticulture and Landscape Gardening, Faculty of Agriculture, Alexandria UniversityAlexandria, Egypt; ^4^Department of Ecology and Evolutionary Biology, University of Connecticut, StorrsCT, United States

**Keywords:** *shadow-1*, transcriptome analysis, differentially expressed genes, dwarfism, gibberellins, shade tolerance

## Abstract

The molecular basis behind shade tolerance in plants is not fully understood. Previously, we have shown that a connection may exist between shade tolerance and dwarfism, however, the mechanism connecting these phenotypes is not well understood. In order to clarify this connection, we analyzed the transcriptome of a previously identified shade-tolerant mutant of perennial ryegrass (*Lolium perenne* L.) called *shadow-1*. *shadow-1* mutant plants are dwarf, and are significantly tolerant to shade in a number of environments compared to wild-type controls. In this study, we treated *shadow-1* and wild-type plants with 95% shade for 2 weeks and compared the transcriptomes of these shade-treated individuals with both genotypes exposed to full light. We identified 2,200 differentially expressed genes (DEGs) (1,096 up-regulated and 1,104 down-regulated) in *shadow-1* mutants, compared to wild type, following exposure to shade stress. Of these DEGs, 329 were unique to *shadow-1* plants kept under shade and were not found in any other comparisons that we made. We found 2,245 DEGs (1,153 up-regulated and 1,092 down-regulated) in *shadow-1* plants, compared to wild-type, under light, with 485 DEGs unique to *shadow-1* plants under light. We examined the expression of gibberellin (GA) biosynthesis genes and found that they were down-regulated in *shadow-1* plants compared to wild type, notably gibberellin 20 oxidase (*GA20ox*), which was down-regulated to 3.3% (96.7% reduction) of the wild-type expression level under shade conditions. One GA response gene, lipid transfer protein 3 (*LTP3*), was also down-regulated to 41.5% in *shadow-1* plants under shade conditions when compared to the expression level in the wild type. These data provide valuable insight into a role that GA plays in dwarfism and shade tolerance, as exemplified by *shadow-1* plants, and could serve as a guide for plant breeders interested in developing new cultivars with either of these traits.

## Introduction

Perennial ryegrass (*Lolium perenne* L.) is one of the most widely cultivated cool-season turfgrass species in the world (Jiang and Huang, 2001; Chen J. et al., 2016). Known for its fast establishment, perennial ryegrass is favored for ornamental use as well as for livestock grazing (Grinberg et al., 2016). While perennial ryegrass is incorporated into many seed mixtures due to its positive traits, it is seldom grown by itself because of its sensitivity to a number of environmental stresses (Gardner and Taylor, 2002; Tegg and Lane, 2004). Perennial ryegrass struggles to grow in overly shady environments, exhibiting shade avoidance response (SAR). This condition is characterized by weak growth, overly elongated leaves, and chlorosis (Franklin and Whitelam, 2005). SAR and other symptoms of shade stress impact virtually all plant taxa, and a high degree of shade has a negative impact on the growth and development of all plants (Nozue et al., 2015). In cereal crop plants, such as maize, shade can inhibit lateral branching, leading to a reduction in overall vegetative biomass (Kebrom et al., 2006; Whipple et al., 2011). Shade has also been shown to reduce the production of grains, such as kernels in maize, as well as seeds, as seen in *Brassica rapa* (Page et al., 2010; Procko et al., 2014).

In seedlings, shade causes etiolation, which is characterized by elongation of the hypocotyls and petioles and, in some cases, the inhibition of cotyledon expansion and reduction in lateral roots (Procko et al., 2014). Petiole elongation is also a symptom of shade stress in adult plants (Kozuka et al., 2005; Sasidharan et al., 2010). These various shade responses are known to be regulated by light-sensing pigments called phytochromes. Shade conditions reduce the activity of phytochromes, of which phytochromes A and B (PhyA, PhyB) have been shown to be especially important. Phytochromes are responsible for repressing the DNA binding activity of phytochrome interacting factors (PIFs; Park et al., 2012). Once free of PhyB repression, PIFs are able to activate various shade-associated physiological responses, such as stem and petiole elongation, through their activity as transcription factors (Lorrain et al., 2008). DELLA proteins, through protein–protein interaction, also repress PIF activity (De Lucas et al., 2008; Feng et al., 2008). DELLA proteins are degraded in the presence of gibberellin (GA) after binding to the GA receptor GID1 via the E3 ubiquitination pathway (Sun, 2008).

In many plant species, shade response is controlled through various phytohormone response pathways, most notably the GA pathway (Yamaguchi, 2008; Colebrook et al., 2014). Previously, we have suggested that GA content has a potential impact on the shade tolerance exhibited by *shadow-1* mutant plants (Li et al., 2016). GAs are terpenoid products, and GA biosynthesis begins when geranylgeranyl pyrophosphate (*GGPP*) is catalyzed into ent-copalyl pyrophosphate by ent-copalyl diphosphate synthase. This product is then modified by a number of upstream biosynthesis enzymes, namely: ent-kaurene synthase (*KS*), ent-kaurene oxidase (*KO*), and ent-kaurenoic acid oxidase (*KAO*). The final steps of bioactive GA biosynthesis are catalyzed by gibberellin 20 oxidase (*GA20ox*) and gibberellin 3 oxidase (*GA3ox*). The process of deactivation of bioactive GAs is governed by gibberellin 2 oxidase (*GA2ox*; Hedden and Phillips, 2000; Chen S. et al., 2016).

In congested areas, whether with buildings in urban areas or with trees in rural areas, it can be difficult to find growing space with adequate light exposure for ornamental plants (Pons and Poorter, 2014). Understanding the mechanisms behind shade tolerance would make it possible to develop solutions to the challenges of growing plants in low-light environments. *shadow-1* is a dwarf, shade-tolerant perennial ryegrass mutant. When subjected to severe shade stress (95% light reduction) *shadow-1* plants are significantly resistant to SAR (Li et al., 2016). The *shadow-1* mutant line represents a valuable opportunity to study the shade response pathway in monocots.

In an attempt to uncover the genetic mechanisms behind dwarfism and shade tolerance in *shadow-1* perennial ryegrass, we have treated *shadow-1* and wild-type plants with 95% shade and compared their transcriptomes to plants kept under full light. Through examination of differential gene expression within the GA biosynthesis and response pathways of *shadow-1* mutant plants, we have implicated decreases in GA content as a likely mechanism for shade tolerance, as well as dwarfism, in these plants. These results provide some insight into the role that GAs may play in shade response, as well as possible strategies for breeding shade tolerant crop plants.

## Materials and Methods

### Plant Treatment and Tissue Sampling

*shadow-1* and wild-type plants were vegetatively propagated in rectangular pots (15 cm × 11 cm × 5 cm). Plant roots and shoots were cut to 2.5 cm and six groups of two tillers were evenly spread within each pot. Plants were maintained at a 5 cm height in full light for 6 weeks. Individuals selected for shade-stress treatment were placed in a 95% shade environment in the greenhouse, which was created by the use of black polyfiber cloth. Those selected for full-sunlight treatment were left out in the open in the greenhouse. After growing for an additional 2 weeks under either light or 95% shade, leaf tissue was collected from six pots (one biological replicate) for each genotype (wild type or *shadow-1*) under each treatment (light or shade). A total of three replicates were collected for each genotype under each treatment. Tissue was collected by cutting young leaves directly into a beaker of liquid nitrogen in an effort to preserve mRNA. For shade-treated plants, this was done in a darkroom environment to avoid light contamination.

### RNA Extraction and Library Preparation

Total plant RNA was extracted using the RNeasy Plant Mini Kit, including RNase-Free DNase set (Qiagen, Valencia, CA, United States), according to the manufacturer’s protocol. RNA purity and concentration were measured using the NanoDrop 2000 spectrophotometer (Thermo Fisher Scientific, Waltham, MA, United States). To further assess RNA quality, total RNA was analyzed on the Agilent TapeStation 2200 (Agilent Technologies, Santa Clara, CA, United States) using the RNA High Sensitivity assay. Ribosomal Integrity Numbers (RINe) were recorded for each sample. Only samples with RINe values above 7.0 were used for library preparation. Total RNA samples were prepared for mRNA-Sequencing using the Illumina TruSeq Stranded mRNA Sample Preparation kit following the manufacturer’s protocol (Illumina, San Diego, CA, United States). Libraries were validated for length and adapter dimer removal using the Agilent TapeStation 2200 D1000 High Sensitivity assay (Agilent Technologies, Santa Clara, CA, United States) and were then quantified and normalized using the dsDNA High Sensitivity Assay for Qubit 2.0 (Life Technologies, Carlsbad, CA, United States). Libraries were prepared for the Illumina HiSeq 2500 (v.4 chemistry) in High Output mode (2 × 100 bp). A total of 12 libraries were sequenced across two lanes.

### Differential Expression Analysis and Functional Annotation

Clean reads were obtained by first removing adapter sequences, and then filtering out reads with over 20% low-*Q*-value (≤20) bases, as well as reads with more than 5% ambiguous “N” bases. The clean reads were then aligned to the perennial ryegrass genome assembled by Byrne et al. (2015) using default parameters in Tophat2 software (Kim et al., 2013). Gene expression levels were calculated as reads per kilobase of transcript per million mapped reads (RPKM). Differentially expressed genes (DEGs) were defined as genes having a false discovery rate (FDR) ≤0.05 and an absolute log_2_ fold change value ≥1. To further characterize the function of DEGs, they were mapped to Gene Ontology (GO) classifications using Blast2GO (Conesa and Götz, 2008). Three categories of GO annotations were analyzed: biological process, molecular function, and cellular component. To uncover GA biosynthesis genes and GA response gene of perennial ryegrass, BLASTP was performed against the translated perennial ryegrass reference genome for each gene of interest. The top hits with an *E*-value <10^-4^ were aligned using ClustalX 2.0 (Larkin et al., 2007). A phylogenetic tree was constructed for all selected hits by PHYML version 3.0 using the maximum likelihood method (Guindon et al., 2010) under the JTT evolutionary model. The closest neighbor for each protein was designated as the putative homolog for that protein in perennial ryegrass. A representative phylogenetic tree was provided in the Supplementary File 1.

### Quantitative Real-Time PCR Analysis

Three genes: *KS*, *KAO*, and *GA20ox* were analyzed using quantitative real-time PCR (qRT-PCR). New plant material was harvested, and RNA was extracted, as previously described. The iScript^TM^ cDNA Synthesis Kit (Bio-Rad Laboratories, Richmond, CA, United States) was used to synthesize cDNA, and cDNA products were utilized for qRT-PCR assays using SsoFast^TM^ EvaGreen^^®^^ Supermix (Bio-Rad Laboratories, Richmond, CA, United States) on a CFX96^TM^ Real-Time PCR detection system (Bio-Rad Laboratories, Richmond, CA, United States). Primer sequences for all genes analyzed are as follows: *KS* forward: 5′-GGAAACCTGCTAGACTGGAA-3′, *KS* reverse: 5′-ATTTAGGTACCCGAGGGCTT-3′, *KAO* forward: 5′-CAGGAAGATGGAGTACCTCT-3′, *KAO* reverse: 5′-ATGTGCACAGTCCTGTACCA-3′, *GA20ox* forward: 5′-GACTTCACGCAGAAGCACTA-3′, *GA20ox* reverse: 5′-GCAGATGCAGAGAAGCAGAA-3′, *LpGAPDH* forward: 5′-CATCACCATTGTCTCCAACG-3′, *LpGAPDH* reverse: 5′-AACCTTCAACGATGCCAAAC-3′. The native glyceraldehyde-3-phosphate dehydrogenase (*LpGAPDH*) gene was used as the internal control (Petersen et al., 2004; Kovi et al., 2016). Data were analyzed using CFX Manager^TM^ software version 2.0. The expression levels in each sample was normalized using the expression level of *LpGAPDH* gene in the same sample. Three biological replicates were performed with each type of sample. Means of gene expression levels between *shadow-1* and wild type were compared using the two-tailed Student’s *t*-test with the pooled variance (Steel et al., 1997).

## Results

### Sequencing and Mapping of the *shadow-1* Transcriptome

**FIGURE 1:**
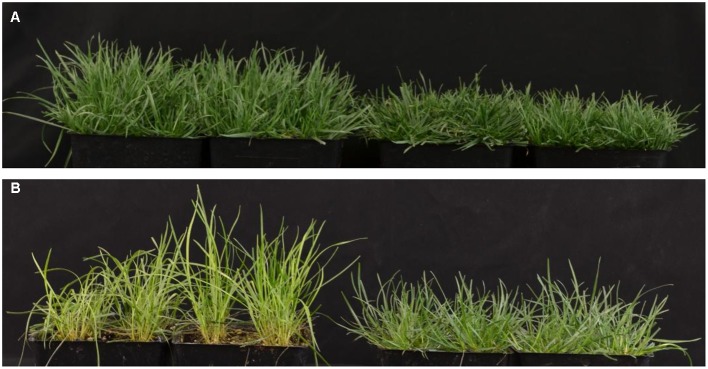
***shadow-1* plants exhibit a dual phenotype of dwarfism and shade tolerance. (A)** Eight-week-old, wild type (left) and *shadow-1* plants (right) grown under full light in the greenhouse. **(B)** Wild type (left) and *shadow-1* plants (right) after 2 weeks under 95% shade in the greenhouse.

When grown in the greenhouse under full light conditions, *shadow-1* plants exhibited dwarfism, categorized by reduced canopy heights compared to wild type (**Figure 1A**). Following 2 weeks of shade treatment, *shadow-1* plants were found to be more tolerant to shade compared to wild type, as evidenced by a significant reduction in leaf elongation and the retention of a healthy, green appearance (**Figure 1B**). These results are consistent with previously reported analysis of the *shadow-1* mutant line (Li et al., 2016).

Following the 2 weeks of shade treatment, leaf tissue samples were harvested from the *shadow-1* and wild-type plants, kept under both shade and light conditions, for transcriptome analysis. We used three biological replicates for each genetic background under each treatment. Transcriptome sequencing data were deposited in the NCBI SRA database under the accession number SRP102018. Through sequencing, we generated a total of 657,122,180 raw reads and 633,014,566 clean reads. The average Q20 and Q30 scores for clean reads among all 12 samples were 95.88 and 90.40%, respectively. For these reads, the average GC content was 50.42% (**Table 1**). We used the perennial ryegrass genome assembled by Byrne et al. (2015) as a reference against which the clean reads from each sample were mapped. We were able to map around 75% of the clean reads for each sample group to the reference genome (**Table 2**).

**Table 1:** Summary of sequencing quality.

Sample	Raw reads	Clean reads	Q20 (%)	Q30 (%)	GC (%)
WT-light-1	58,808,386	57,391,990	95.93	90.95	51.07
WT-light-2	55,033,008	53,234,472	95.81	90.43	50.92
WT-light-3	56,770,090	54,313,758	95.92	90.28	50.40
*shadow-1*-light-1	55,896,890	53,896,162	95.96	90.56	50.22
*shadow-1*-light-2	59,597,674	57,352,466	95.92	90.48	50.06
*shadow-1*-light-3	58,432,782	55,603,944	95.88	89.97	49.71
WT-shade-1	52,736,878	50,437,952	95.81	89.99	50.66
WT-shade-2	50,532,670	49,147,900	95.89	90.75	50.59
WT-shade-3	55,565,928	53,980,982	96.11	91.02	50.47
*shadow-1*-shade-1	56,892,916	55,026,380	95.86	90.52	50.60
*shadow-1*-shade-2	48,139,324	46,160,156	95.95	90.37	50.49
*shadow-1*-shade-3	48,715,634	46,468,404	95.57	89.52	49.90
Total	657,122,180	633,014,566			


*Q20, percentage of bases with a Phred value >20; Q30, percentage of bases with a Phred value >30*.

**Table 2:** Clean reads were mapped at high percentage to the perennial ryegrass genome.

Sample group	Total clean reads	Mapped reads (%)
wild type light	164,940,220	75.80
*shadow-1* light	166,852,572	75.18
wild type shade	153,566,834	74.84
*shadow-1* shade	147,654,940	76.64


**FIGURE 2:**
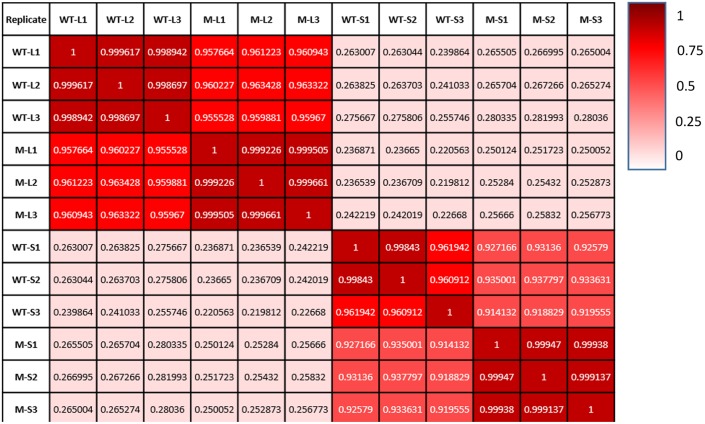
**Each sample type shows a high degree of consistency between replicates.** Comparisons of gene expression between replicates on the *x*-axis and those on the *y*-axis. Pearson correlation coefficient, as well as a color value (whiter—less similar; redder—more similar), are given for each comparison.

We have compared gene expression among the three biological replicates for all four sample types: wild type kept under light, *shadow-1* kept under light, wild type treated with shade, and *shadow-1* treated with shade. The similarity of expression profiles between the replicates was determined by a Pearson correlation coefficient analysis. We found that the three biological replicates for each sample type were highly correlated (*r* > 0.96), demonstrating consistency between replicates regarding to DEGs (**Figure 2**).

### Differential Gene Expression

**FIGURE 3:**
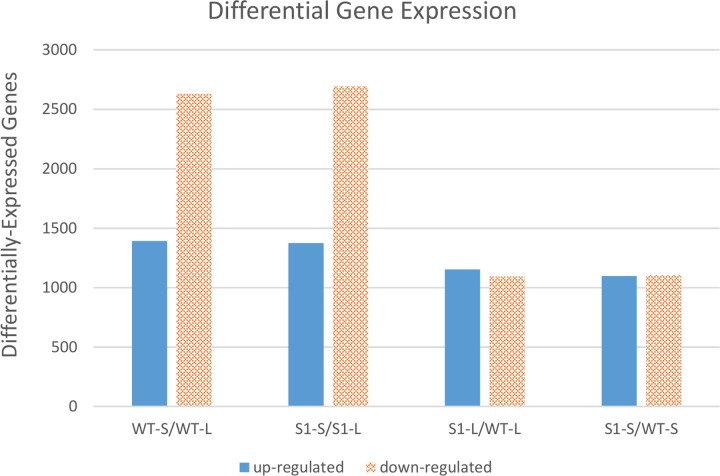
**Comparisons of differentially expressed genes between *shadow-1* and wild type under light and shade.** Each pair of bars represents a comparison between two sample types. WT-L, wild type plants kept under full light; WT-S, wild type plants treated with 95% shade; *S1*-L, *shadow-1* plants kept under full light; *S1*-S, *shadow-1* plants treated with 95% shade.

When we examined the number of DEGs for each of these four comparisons (**Figure 3**), we observed that shade treatment caused more changes in gene expression (i.e., more DEGs) than the mutation(s) in *shadow-1* plants under either light or shade conditions. These results demonstrate that shade stress has a larger impact on gene expression than the mutation(s).

**FIGURE 4:**
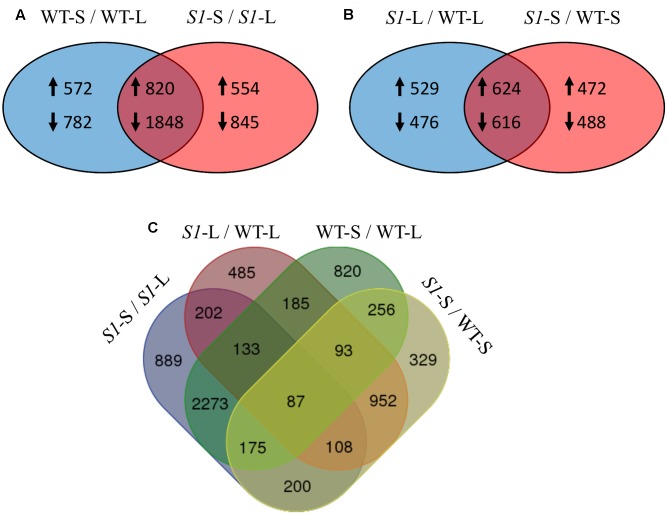
**Overlapping of differentially expressed genes across treatments (shade vs full light) and genotypes (wild type vs *shadow-1*). (A)** Comparison between DEGs identified in wild type following shade treatment (left), to those identified in *shadow-1* following shade treatment (right). The overlapping region represents DEGs shared between *shadow-1* and wild type following shade treatment. **(B)** Comparison between DEGs identified in *shadow-1* (vs wild type) under light (left) to those identified following shade treatment (right). The overlapping region represents DEGs shared between *shadow-1* plants kept under light and those treated with shade, compared to wild type under the same conditions. **(A,B)** Up-arrows signify up-regulated DEGs and down-arrows signify down-regulated DEGs. **(C)** Four-way Venn figure including all comparisons from **(A)** and **(B)**. WT-L, wild type plants kept under full light; WT-S, wild type plants treated with 95% shade; *S1*-L, *shadow-1* plants kept under full light; *S1*-S, *shadow-1* plants treated with 95% shade.

There were 4,022 DEGs in shade-treated wild-type plants, compared to those which were grown in the light, with 1,392 up-regulated and 2,630 down-regulated. Similarly, there were 4,067 DEGs (1,374 up-regulated and 2,693 down-regulated) in shade-treated *shadow-1* plants compared to those kept under light (**Figure 3**). 2,668 DEGs (820 up-regulated and 1,848 down-regulated) were shared between shade-treated *shadow-1* and shade-treated wild-type plants, when each were compared to their light-grown counterparts (**Figure 4A**). It is likely that many of these genes are not involved in the shade tolerance exhibited by *shadow-1* plants, but instead are representative of the general shade response of perennial ryegrass.

There were 2,245 DEGs (1,153 up-regulated and 1,092 down-regulated) uncovered in light-grown *shadow-1* plants, compared to wild-type and 2,200 DEGs (1,096 up-regulated and 1,104 down-regulated) uncovered in shade-treated *shadow-1* plants, compared to wild type (**Figure 3**). There were 1,240 DEGs (624 up-regulated and 616 down-regulated) shared by *shadow-1* plants under light and shade, when each were compared to wild-type under the same conditions. 1,005 DEGs (529 up-regulated, 476 down-regulated) were found only in light-grown *shadow-1* (compared to wild type) and 960 DEGs (472 up-regulated, 488 down-regulated) found only in shade-treated *shadow-1* (compared to wild type) (**Figure 4B**).

We also compared differential gene expression between light-grown *shadow-1*, shade-treated *shadow-1*, light-grown wild-type, and shade-treated wild type, in a four-way comparison (**Figure 4C**). This four-way comparison exposed 329 DEGs that were unique to *shadow-1* when compared to wild-type under shade conditions, and 485 DEGs that were unique to *shadow-1* if compared to wild type under light conditions. There were also 820 DEGs that were unique to shade-treated wild-type plants (compared to wild type under light), and 889 DEGs that were unique to shade-treated *shadow-1* plants (compared to *shadow-1* under light). There were 87 DEGs that had differential expression in *shadow-1* and wild type under both light and shade.

**FIGURE 5:**
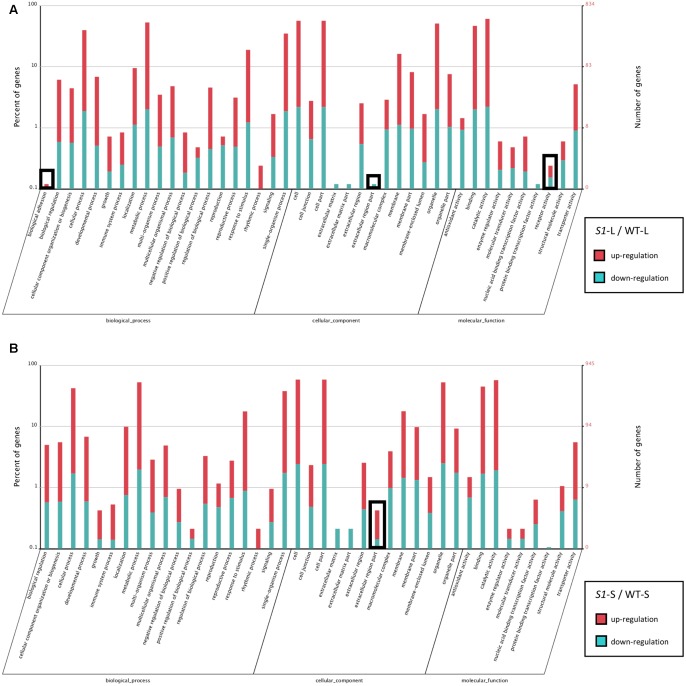
**Functional gene classification of DEGs. (A)** Gene Ontology (GO) distribution of DEGs identified in *shadow-1* plants compared to wild type kept under full light. **(B)** GO distribution of DEGs identified in *shadow-1* plants compared to wild type after shade treatment. Black boxes highlight differences between specific terms in **(A)** and **(B)**. *S1*-L, *shadow-1* plants kept under full light; WT-L, wild type plants kept under full light; *S1*-S, *shadow-1* plants treated with 95% shade; WT-S, wild type plants treated with 95% shade.

In order to explore the function of DEGs identified in *shadow-1* plants (compared to wild type) under both light and shade conditions, we performed GO enrichment analysis. The enriched GO distributions were similar for *shadow-1* plants under light and under shade (compared to wild type under the same conditions), however, there were a few notable differences. We examined some of the genes from the GO groups that showed differences and queried the associated proteins against the NCBI NR protein database. DEGs involved in “biological adhesion” and “receptor activity” for *shadow-1* plants kept under light were absent in *shadow-1* plants treated with shade (**Figure 5**). The “biological adhesion” group included a gene that coded for ERECTA, a receptor-like kinase, and was up-regulated (9.85×) in *shadow-1* plants compared to wild type under light. The “receptor activity” group included a gene that coded for PhyA, a light receptor, was also up-regulated (7.24×) in *shadow-1* plants under light. For another GO group, “extracellular region part,” *shadow-1* plants under light had only down-regulated DEGs, while shade-treated *shadow-1* plants had both up- and down-regulated DEGs (**Figure 5**). One gene within this group coded for cytokinin oxygenase, which degrades bioactive cytokinin, and was up-regulated (11.38×) in *shadow-1* plants under shade compared to wild type, but was not differentially regulated in *shadow-1* under light.

### DEGs in the GA Pathway

**FIGURE 6:**
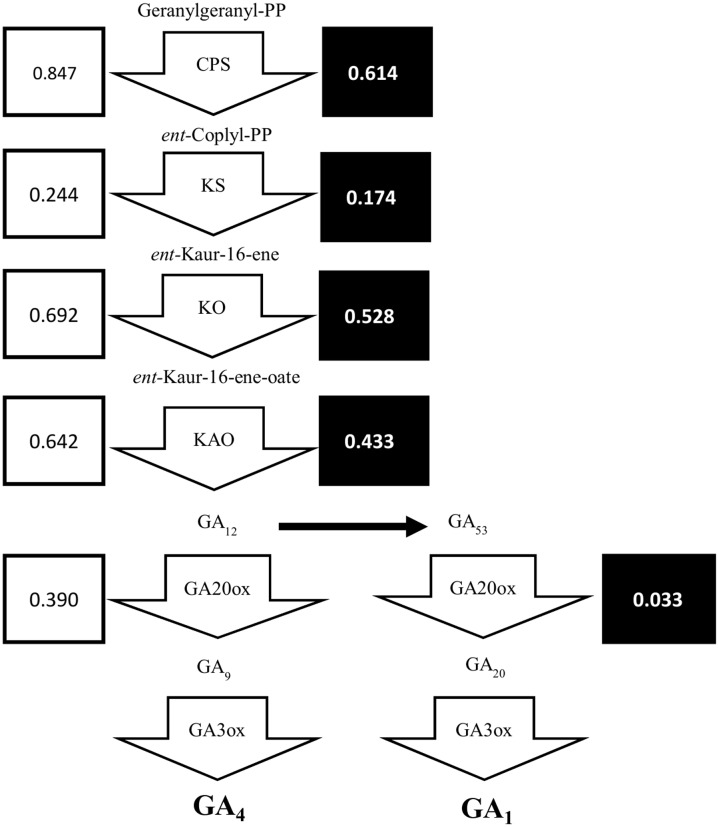
**The gibberellin biosynthesis pathway was down-regulated in *shadow-1*.** Arrow boxes represent areas of the pathways catalyzed by specific proteins. Unboxed text represents terpene products at each step in GA biosynthesis pathway. White boxes to the left of each gene show the expression for each gene in *shadow-1* plants under light, compared to wild-type plants under the same conditions, with wild-type expression normalized to 1. Black boxes to the right of each gene show the expression for each gene in *shadow-1* plants treated with shade stress, compared to wild-type plants under the same conditions, with wild-type expression normalized to 1. CPS, ent-copalyl diphosphate synthase; KS, ent-kaurene synthase; KO, ent-kaurene oxidase; KAO, ent-kaurenoic acid oxidase.

Previously, we showed that the dwarfism and shade-tolerance phenotypes displayed in *shadow-1* might be caused by changes in GA concentration (Li et al., 2016). The genes responsible for GA biosynthesis are poorly annotated in perennial ryegrass, but are well characterized in bread wheat (*Triticum aestivum*), a close relative of perennial ryegrass. To uncover DEGs within the GA biosynthesis pathway, we selected the protein sequences for enzymes catalyzing key steps of GA biosynthesis in bread wheat and aligned them to the translated perennial ryegrass reference genome (Supplementary File 2). As shown in **Figure 6**, putative GA biosynthesis genes were down-regulated in *shadow-1* plants (compared to wild type) under both light and shade conditions. Under light, the GA biosynthesis genes, *CPS*, *KS*, *KO*, and *KAO*, were down-regulated to 24.4–84.7% of the levels of wild-type plants. Under shade conditions, these genes were also down-regulated to 17.4–61.4% of the wild type plants. The downstream GA biosynthesis splits into two pathways, one for GA_1_ and another for GA_4_. Both pathways are catalyzed by *GA20ox* followed by *GA3ox*, which are responsible for key steps in GA biosynthesis. Expression of *GA20ox* was reduced in *shadow-1* plants kept under light, to 39.0% of the expression in wild type. Under the shade conditions, the expression of *GA20ox* in *shadow-1* plants was reduced to 3.3% when compared to the wild type control. We were unable to uncover putative homologs of *GA3ox* through annotation.

**FIGURE 7:**
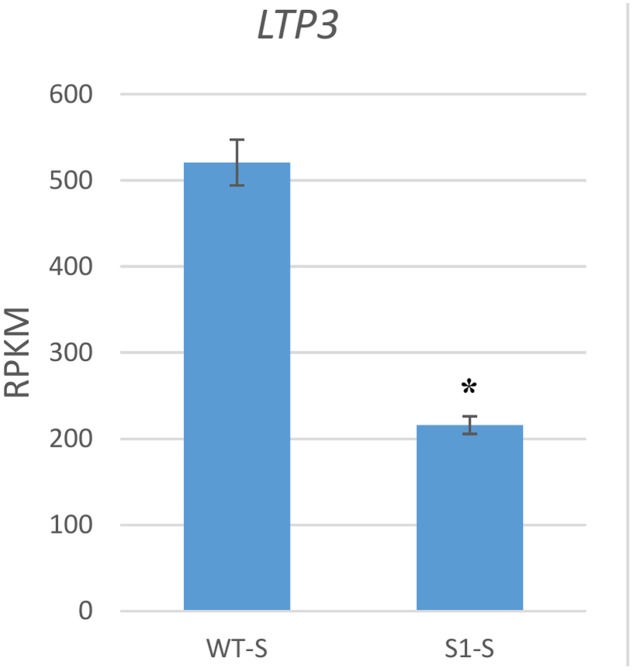
**GA response gene was down-regulated in *shadow-1* under shade.** Expression level of the GA response gene *LTP3* was significantly decreased in shade-treated *shadow-1* plants, compared to wild-type under the same conditions. Gene expression levels were calculated using reads per kilobase of transcript per million mapped reads (RPKM) values. Data represent means from three independent biological replicates. Bars show standard errors. Asterisk represents a significant difference when compared to wild type under same conditions using two-tailed Student’s *t*-test (*p* ≤ 0.05). WT-S, wild type plants treated with shade; *S1*-S, *shadow-1* plants treated with shade.

We next examined one GA response gene, lipid transfer protein 3 (*LTP3*), which is up-regulated by GA (De Lucas et al., 2008). *LTP3* was down-regulated to 41.5% in *shadow-1* plants if compared to its expression in wild type under shade conditions (**Figure 7**). This gene was also down-regulated in *shadow-1* plants when comparing to the wild-type under nature light (data not shown).

### Verification of Differentially Expressed Genes via qRT-PCR

**FIGURE 8:**
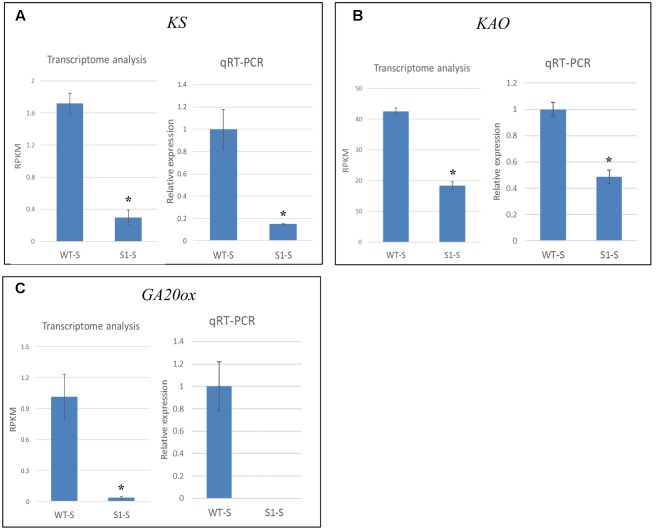
**qRT-PCR data verified the accuracy of transcriptome analysis.** Expression levels of *KS* **(A)**, *KAO* **(B)**, and *GA20ox* **(C)** were identified through transcriptome and qRT-PCR analyses. For transcriptome analysis, gene expression levels were calculated using reads per kilobase of transcript per million mapped reads (RPKM) values. For qRT-PCR, the gene expression levels in each sample were normalized using the expression level of the internal control, *LpGAPDH*, in the same sample, and wild-type expression levels were normalized to 1. The data presented are the means from three independent biological replicates. Bars show standard errors. Asterisk represents a significant difference when compared to wild type under same conditions using two-tailed Student’s *t*-test (*p* ≤ 0.05). WT-S, wild type plants treated with shade; *S1*-S, *shadow-1* plants treated with shade.

We verified the accuracy of our transcriptome data by selecting three genes (*KS*, *KAO*, and *GA20ox*) for qRT-PCR analysis, using mRNA extracted from shade-treated wild-type and *shadow-1* plants. The results of qRT-PCR analysis showed similar expression patterns to those obtained from our transcriptome analysis (**Figure 8**). Transcriptome analysis demonstrated that, in shade-treated *shadow-1*, *KS* expression was reduced to 17.4% of its expression in wild type, while qRT-PCR showed down-regulation to 14.8% of wild-type expression. Under the same conditions, transcriptome analysis showed that *KAO* was down-regulated in *shadow-1* to 43.3% of its wild-type expression, while qRT-PCR showed down-regulation to 49.7%. For shade-treated *shadow-1*, *GA20ox* was down-regulated to 3.3% of its expression in wild type. 3.3% represents a total of only 0.03 mapped reads (RPKM) over millions of sequencing reactions, which is in the barely detectable level. Consistently, we could not detect expression of this gene for these plants with our qRT-PCR analysis. In summary, the expression patterns detected with the transcriptome and qPCR analyses are consistent in general, demonstrating that the transcriptome data from Illumina sequencing analysis are reliable.

## Discussion

In this study, we analyzed the transcriptomes of the shade-tolerant perennial ryegrass mutant *shadow-1* kept under light and shade, to explore the mechanisms behind both dwarfism and shade-tolerance in these plants. We discovered that, in the light, there were noteworthy differences in gene expression between the *shadow-1* mutant and wild type, in the form of 2,245 DEGs. There were similar differences in gene expression between these two genotypes after both were subjected to shade stress (2,200 DEGs). When we compared DEGs across genotypes and across treatments, there were 485 DEGs that were unique to *shadow-1* (compared to wild type) under light, and there were 329 DEGs that were unique to *shadow-1* (compared to wild type) under shade. Additionally, we uncovered 87 DEGs which were differentially expressed in *shadow-1* (compared to wild type) under light and shade, and were also differentially expressed in wild type under shade (compared to wild type under light) and *shadow-1* under shade (compared to *shadow-1* under light). Furthermore, we have observed an overall down-regulation of GA biosynthesis genes in *shadow-1* plants compared to wild type, under both light and shade conditions, most notably *GA20ox*, which was down-regulated to 3.3% in *shadow-1* plants under shade conditions. These data provide additional support for our hypothesis (Li et al., 2016) that GA plays a key role in both dwarfism and shade tolerance, as shown by *shadow-1* plants.

Our results have shown that key GA biosynthesis genes, as well as one GA response gene, were down-regulated in the *shadow-1* transcriptome compared to wild type, under both light and shade conditions. We also checked the expression of *GID1*, the main receptor of GA, and found there was no difference in expression at mRNA level between *shadow-1* and wild type under either light or shade conditions. Evidence pointing toward decreased GA biosynthesis in *shadow-1* plants is consistent with our previous report that both dwarfism and shade tolerance in *shadow-1* plants can be abolished through exogenous application of gibberellic acid (GA3; Li et al., 2016). We have also previously shown that interruption of GA biosynthesis, through the application of trinexapac-ethyl (TE) to wild-type plants, is sufficient to cause dwarfism and shade tolerance in these plants. TE acts by disrupting the latter steps of GA biosynthesis, such as those controlled by *GA20ox* (Hedden and Thomas, 2012), which has now been shown to be down-regulated in *shadow-1* plants under both light and shade conditions. Our data suggest that dwarfism and shade tolerance are connected in *shadow-1* through the activity of GA. If this is the case, it is likely that dwarfism, through the mechanism of reduced leaf elongation, provides tolerance to shade stress.

Through Illumina sequencing, we generated millions of clean reads, representing gigabytes of sequencing data, which were efficiently mapped to a reference genome. There was high consistency between the biological replicates used for RNA sequencing (*r* ≥ 96%), demonstrating the reliability of the data produced. We were also able to confirm the accuracy of our transcriptome analysis via qRT-PCR analysis. Together, these data demonstrate the excellent reproducibility of the results. The transcriptome data acquired from *shadow-1* plants under light and shade conditions are valuable resources for exploring genetic mechanisms underlying dwarfism and shade tolerance.

We were unable to pinpoint the exact gene(s) whose mutation was responsible for the mutant phenotypes exhibited by *shadow-1* plants. However, these mutant genes cause downstream changes in gene expression that are observable through our transcriptome analysis. If the dwarf and shade-tolerant phenotypes exhibited by *shadow-1* plants are caused by the same mutation(s), the DEGs resulting from the mutation(s) should be shared by *shadow-1* under light and *shadow-1* under shade (compared to wild type under the same conditions). Because of the down regulation of the GA biosynthesis genes observed in the *shadow-1* mutant plants, one possibility is that a key gene such as a transcription factor gene involved in regulating the overall GA biosynthetic pathway may be knocked out. Further characterization of the mutant plants is needed to address these questions.

As was shown in our GO enrichment analysis, there were differences in the gene expression of *shadow-1* plants (compared to wild type), depending on whether plants were kept under light or were treated with shade, which provides insight into potential differences between dwarfism and shade tolerance for these plants. We identified three of these genes, coding for ERECTA (up-regulated in *shadow-1* in light), PhyA (up-regulated in *shadow-1* in light), and cytokinin deoxygenase (up-regulated in *shadow-1* in shade). ERECTA and cytokinin deoxygenase are both related to cell division/proliferation (Riou-Khamlichi et al., 1999; Shpak et al., 2004), which could have an impact on dwarfism and shade tolerance by influencing leaf elongation. Leaf elongation is associated with GA response, especially under low-light conditions (Tan and Qian, 2003; Xu et al., 2016), making it likely that the expression of these two genes have some connection to downstream GA signaling. Additionally, phytochrome is indirectly involved in the GA response pathway through the activity of PIFs, which act as transcription factors and have been implicated in the GA-mediated light response (Alabadí et al., 2008; Lorrain et al., 2008; Shin et al., 2009). It would be interesting to further dissect the role of these genes in shade responses of turf grasses.

The *shadow-1* mutant line may serve as a good model plant for the study of mechanisms leading to dwarfism and shade tolerance in plants. Both of these traits have utility for plant breeders, in areas ranging from agricultural to ornamental (Wilkins, 1991). Dwarf plants can have increased crop yields and could have reduced requirements for nutrients (Monna et al., 2002). Shade-tolerant plants are able to thrive in environments that are traditionally unconducive to healthy plant growth, such as under tree canopies or in dense urban areas (Jiang et al., 2004). Our transcriptome analysis suggests that, in *shadow-1* plants, the genetic mechanism of both dwarfism and shade tolerance is the down-regulation of genes across the GA biosynthesis pathway. This information could be valuable to turf geneticists and breeders who are interested in developing new cultivars that have either, or both, of these traits.

## Author Contributions

WL and LK-G are equally contributed and did most of the work for mutant characterization. XG and XW were responsible for mapping of transcriptome data. WL, LK-G, XG, XW, and RE-T were responsible for analysis of DEGs. RE-T and HY were also involved greenhouse and field evaluation of the mutant. CT was involved in gamma-ray irradiation and producing M2 seeds and initial screening of the mutant. JI, KG, and RM provided advices on characterization of the mutant and manuscript editing. JW provided advices on transcriptome data analysis and manuscript editing. YL and TG designed experiments for the mutant characterization and transcriptome analysis. WL, LK-G, and YL were involved in manuscript writing and editing.

## Conflict of Interest Statement

The authors declare that the research was conducted in the absence of any commercial or financial relationships that could be construed as a potential conflict of interest.
