# Supplementary material for: Transcriptome Analysis Reveals Differential Gene Expression and a Possible Role of Gibberellins in a Shade-Tolerant Mutant of Perennial Ryegrass
Source: Front Plant Sci. 2017 May 26;8:868. doi: 10.3389/fpls.2017.00868 (PMC5445233; doi:10.3389/fpls.2017.00868)
Supplement: Supplementary file 1 [file Data_Sheet_1.docx]

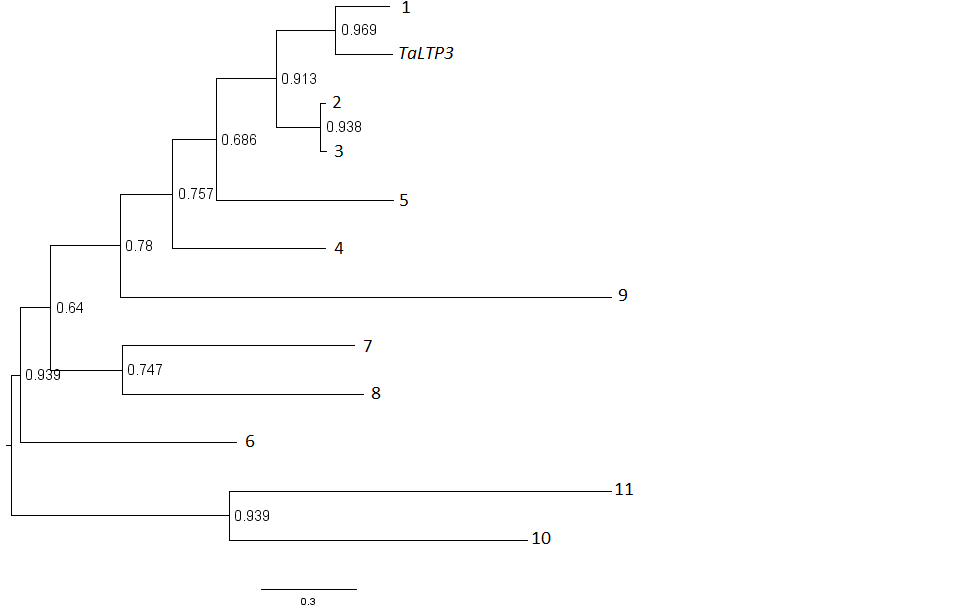


**1.** scaffold_12948_ref0040030-exonerate_est2genome-gene-0.0

**2.** scaffold_22200_ref0020751-exonerate_est2genome-gene-0.0

**3.** scaffold_4353_ref0033990-exonerate_est2genome-gene-0.2

**4.** scaffold_2094_ref0032586-exonerate_est2genome-gene-0.1

**5.** scaffold_17042_ref0036662-exonerate_est2genome-gene-0.0

**6.** scaffold_1962_ref0037401-exonerate_est2genome-gene-0.2

**7.** scaffold_8708_ref0036894-exonerate_est2genome-gene-0.2

**8.** scaffold_3427_ref0047537-exonerate_est2genome-gene-0.3

**9.** scaffold_16892_ref0018604-exonerate_est2genome-gene-0.0

**10.** scaffold_858_ref0017047-exonerate_est2genome-gene-0.6

**11.** scaffold_5761_ref0041974-exonerate_est2genome-gene-0.0

**Supplementary File 1 | A phylogenetic tree to uncover** **homolog of *TaLTP3* in perennial ryegrass.** The GA response gene, *TaLTP3* (Lipid Transfer Protein 3 in *T. aestivum*), was used to blast against the translated perennial ryegrass reference genome. A phylogenetic tree was constructed for all 11 hits with a E-value < 10^-4^ using the Maximum Likelihood method (Guindon et al., 2010) under the JTT evolutionary model. The scale shows the estimated branch length corresponding to the number of substitutions per site. The closest relative (scaffold_12948_ref0040030-exonerate_est2genome-gene-0.0) to the query protein sequence on the phylogenetic tree was considered as the putative homolog in perennial ryegrass.

| Gene name | Scaffold name |
| --- | --- |
| *CPS* | scaffold_7692_ref0045474-exonerate_est2genome-gene-0.1 |
| *KS* | scaffold_3089_ref0029016-exonerate_est2genome-gene-0.1 |
| *KO* | scaffold_2519_ref0039696-exonerate_est2genome-gene-0.1 |
| *KAO* | scaffold_3324_ref0032628-exonerate_est2genome-gene-0.0 |
| *GA20ox* | scaffold_902_ref0007605-exonerate_est2genome-gene-0.0 |
| *LTP3* | scaffold_12948_ref0040030-exonerate_est2genome-gene-0.0 |

**Supplementary File 2 | Scaffold names of the homologs of GA biosynthesis and GA response genes used in this paper.**
